# Increased Expression of the Very Low-Density Lipoprotein Receptor Mediates Lipid Accumulation in Clear-Cell Renal Cell Carcinoma

**DOI:** 10.1371/journal.pone.0048694

**Published:** 2012-11-19

**Authors:** Jeanna Perman Sundelin, Marcus Ståhlman, Annika Lundqvist, Max Levin, Paolo Parini, Martin E. Johansson, Jan Borén

**Affiliations:** 1 Department of Molecular and Clinical Medicine/Wallenberg Laboratory, University of Gothenburg, Gothenburg, Sweden; 2 Division of Clinical Chemistry, Department of Laboratory Medicine, Karolinska Institute, Karolinska University Hospital, Huddinge, Stockholm, Sweden; 3 Center for Molecular Pathology, Department of Laboratory Medicine, Lund University, Department of Pathology, Malmö, Sweden; University of Central Florida, United States of America

## Abstract

Clear-cell renal cell carcinoma (RCC) is, in most cases, caused by loss of function of the tumor suppressor gene von Hippel–Lindau, resulting in constitutive activation of hypoxia-inducible factor (HIF)-1α and expression of hypoxia-induced genes in normoxic conditions. Clear-cell RCC cells are characterized histologically by accumulation of cholesterol, mainly in its ester form. The origin of the increased cholesterol remains unclear, but it is likely explained by an HIF-1α-driven imbalance between cholesterol uptake and excretion. Here, we showed that expression of the very low-density lipoprotein receptor (VLDL-R) was significantly increased in clear-cell RCC human biopsies compared with normal kidney tissue. Partial knockdown of HIF-1α in clear-cell RCC cells significantly reduced the VLDL-R expression, and knockdown of either HIF-1α or VLDL-R reduced the increased lipid accumulation observed in these cells. We also showed increased uptake of fluorescently labeled lipoproteins in clear-cell RCC cells, which was significantly reduced by knockdown of HIF-1α or VLDL-R. Taken together, our results support the concept that the pathological increase of HIF-1α in clear-cell RCC cells upregulates VLDL-R, which mediates increased uptake and accumulation of lipids. These results explain the morphological characteristics of clear-cell RCC, and open up novel possibilities for detection and treatment of clear-cell RCC.

## Experiments

The clear-cell form of renal cell carcinoma (RCC) is the most common type of renal malignancy, accounting for approximately 2.5% of all U.S. cancer diagnoses annually [1], [2]. The neoplastic cells of clear-cell RCC are characterized histologically by a distinctive pale, glassy cytoplasm, which results from intracellular storage of lipid and glycogen [3]. In most cases of clear-cell RCC, hypoxia-inducible factor (HIF)-1α is constitutively activated by inactivation or loss of the von Hippel–Lindau (*VHL*) tumor suppression gene [4], [5], [6], [7]. VHL is part of the E3-ubiquitin ligase complex that binds to the HIF-1α subunit under normoxic conditions and directs it to proteolysis. In the absence of functional VHL, HIF-1α is not degraded but translocates to the nucleus to form a dimer with HIF-1β. The dimer then binds to the hypoxia response element (HRE) motif of target genes to activate or suppress their transcription. Thus, in clear-cell RCC, hypoxia-induced genes are expressed even in normoxic conditions.

The most consistently stored lipid in clear-cell RCC cells is cholesterol, primarily in its ester form [8]. It is not clear what causes the excessive accumulation of cholesterol in these cells, but potential explanations include defects in cholesterol synthesis, cholesterol efflux and/or cholesterol uptake. The possibility that excessive *de novo* synthesis of cholesterol is the primary cause of cholesterol accumulation in clear-cell RCC has been excluded in earlier studies that showed lower activity of HMG-CoA reductase (the rate-limiting enzyme in cholesterol synthesis) and reduced cholesterol synthesis in renal cancer cells [8], [9]._ENREF_7 An abnormality in cholesterol efflux from these cells has not been identified, but Gebhard et al. showed increased activity of acyl-CoA:cholesterol acyltransferase (ACAT) in clear-cell RCC cells [8]._ENREF_6 This enzyme catalyzes the intracellular esterification of cholesterol, and thus promotes the channeling of free cholesterol within the tumor cells into storage as cholesteryl esters rather than being released from the cells. Earlier work to investigate if cholesterol uptake is altered in clear-cell RCC compared accumulation of a radioactive cholesterol analog in tumor tissue and normal renal parenchyma and showed no differences [10]. Furthermore, malignantly transformed renal tissue lacks the main receptor for exogenous cholesterol, the low-density lipoprotein receptor (LDL-R) [11]. However, these studies do not exclude the possibility that the lipid accumulation in clear-cell RCC is due to increased uptake of plasma lipoproteins through an alternative receptor.

We have recently elucidated a novel mechanism for hypoxia-induced lipid accumulation in cardiomyocytes and shown that the lipid accumulation in ischemic heart tissue is caused by upregulation of the very low-density lipoprotein receptor (VLDL-R) [12]. The VLDL-R, which shows considerable similarity to the LDL-R, binds and mediates uptake of triglyceride-rich lipoproteins by endocytosis [12], [13], [14]. Hypoxia-induced VLDL-R expression is dependent on HIF-1α through its interaction with a HRE in the *Vldl-r* promoter [12].

**Figure 1:**
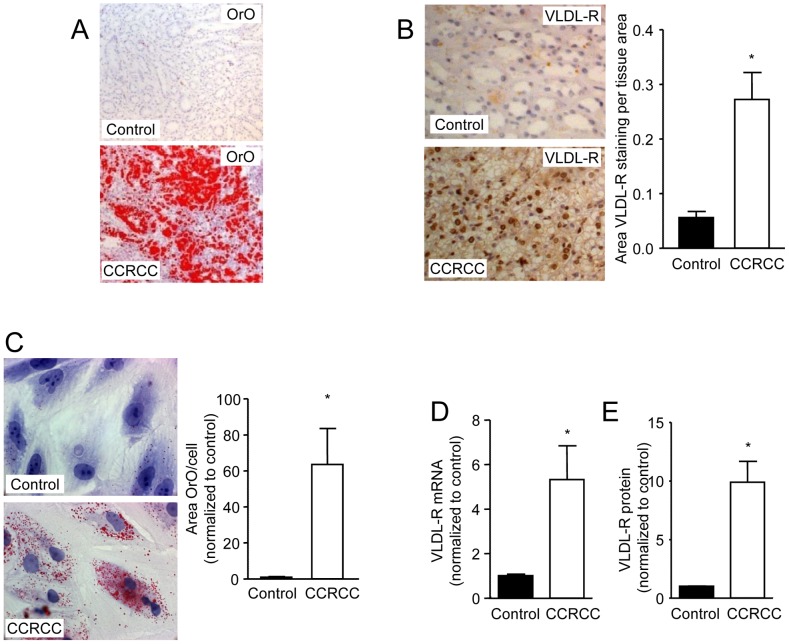
VLDL-R expression is increased in clear-cell RCC. (**A**) Oil Red O staining of human tissue sections from normal kidney tissue (upper) and clear-cell RCC tissue (CCRCC; lower). (**B**) VLDL-R immunostaining (left) and quantification of immunostaining (right) of human tissue sections from normal kidney tissue (upper) and clear-cell RCC tissue (lower) (*n* = 6, *p = 0.0022). (**C**) Oil Red O staining (left) and quantification of staining (right) of cultured human cells isolated from healthy kidney tissue (upper) and clear-cell RCC tissue (lower) (*n* = 10, *p = 0.0058). (**D**) Quantification of VLDL-R mRNA normalized to 18S mRNA from cultured human cells isolated from healthy kidney tissue and clear-cell RCC tissue (*n* = 6, *p = 0.004). (**E**) Quantification of immunoblot against VLDL-R with β-actin as loading control from cultured human cells isolated from healthy kidney tissue and clear-cell RCC tissue (*n* = 6, *p = 0.0002). Data are shown as mean ± SEM.

On the basis of these earlier findings, we hypothesized that lipid accumulation in clear-cell RCC is mediated by overexpression of the VLDL-R. To test this hypothesis, renal cell carcinoma and normal kidney tissue were obtained from nephrectomies from six patients. Lipid accumulation was clearly visible in the clear-cell RCC biopsies but not in the normal kidney tissue (Figure 1A). Lipid analysis showed that the clear-cell RCC biopsies accumulated mainly cholesteryl esters (Table 1) with significantly more cholesteryl oleate (18∶1) and significantly less cholesteryl linoleate (18∶2) than normal kidney tissue (Table S1), in agreement with earlier studies [8]. Importantly, we showed that expression of the VLDL-R protein was four-fold higher in biopsies from clear-cell RCC tissue than in normal control tissue (Figure 1B). We confirmed lipid accumulation in primary cells isolated from clear-cell RCC tissue compared with primary kidney cells isolated from normal control tissue (Figure 1C), and showed significantly higher expression of VLDL-R mRNA (5-fold) and protein (almost 10-fold) in the clear-cell RCC cells compared with control kidney cells (Figure 1D and E).

**Table 1:** Lipid classes in human tissue sections from normal kidney tissue and clear-cell RCC tissue (CCRCC).

Lipid classes	Control	CCRCC	*p* value
	(*n* = 6)	(*n* = 6)	
Cholesteryl ester	0.57±0.24	131±100	0.0088
Free cholesterol	5.2±1.0	8.2±2.3	0.0117
Triacylglycerol	2.2±0.7	20.2±14.2	0.0108
Diacylglycerol	0.071±0.027	0.20±0.13	0.0452
Phosphatidylcholine	5.3±0.6	4.8±0.6	ns
Ether-linked phosphatidylcholine	0.50±0.08	0.66±0.15	0.0354
Phosphatidylethanolamine	2.3±0.2	0.68±0.19	0.0001
Ether-linked phosphatidylethanolamine	0.29±0.02	0.14±0.03	0.0001
Phosphatidylserine	0.15±0.06	0.15±0.07	ns
Lysophosphatidylcholine	0.12±0.01	0.071±0.053	ns
Sphingomyelin	1.4±0.1	1.0±0.2	0.0009
Ceramide	0.036±0.005	0.040±0.011	ns

All values are nmol/mg. Data are mean ± SEM.

**Figure 2:**
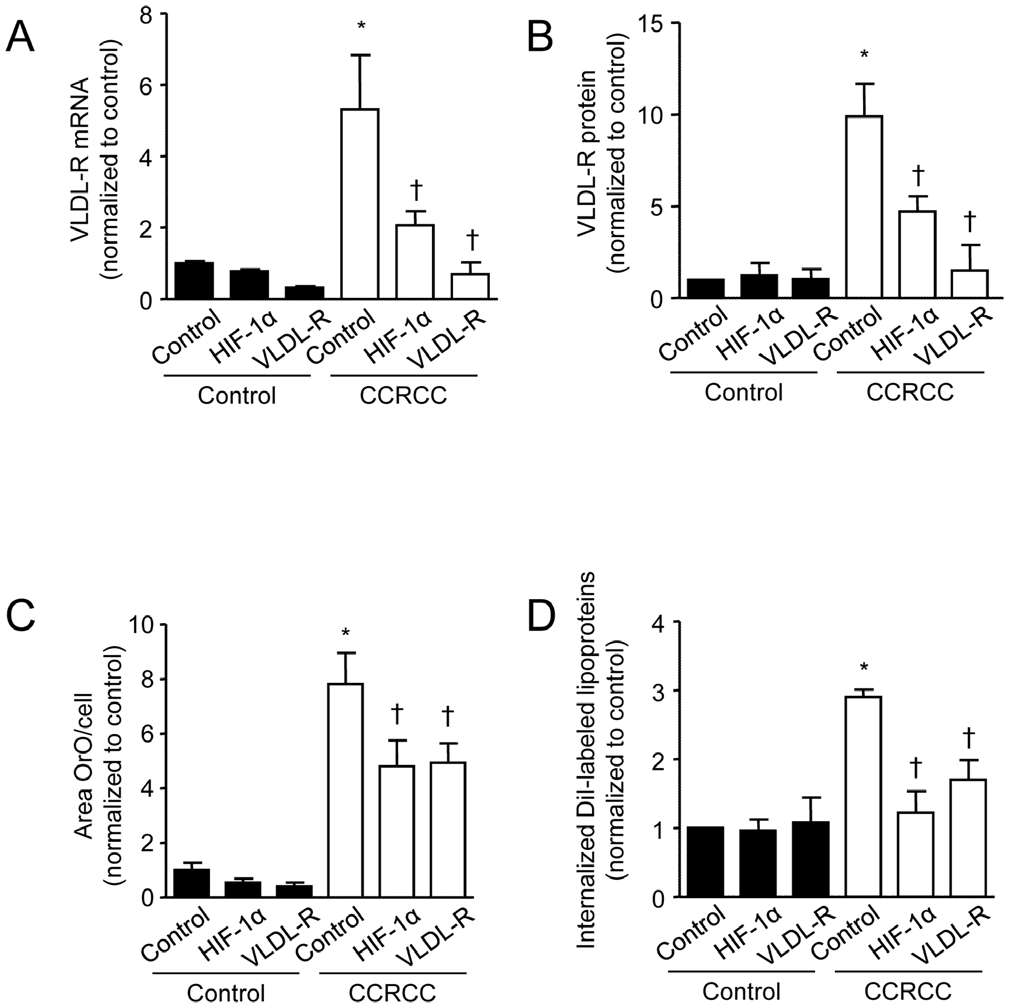
VLDL-R overexpression in clear-cell RCC cells is mediated by HIF-1α, and promotes increased lipid accumulation through increased lipid uptake. (**A**) Quantification of VLDL-R mRNA normalized to 18S mRNA from cultured human cells isolated from healthy kidney tissue and clear-cell RCC tissue treated with siRNA against HIF-1α or VLDL-R (*n* = 10, *p≤0.05 vs. control siRNA normal cells, †*p*≤0.05 vs. control siRNA clear-cell RCC cells). (**B**) Quantification of immunoblot against VLDL-R with β-actin as loading control from cultured human cells isolated from healthy kidney tissue and clear-cell RCC tissue treated with siRNA against HIF-1α or VLDL-R (*n* = 10, *p≤0.05 vs. control siRNA normal cells, †*p*≤0.05 vs. control siRNA clear-cell RCC cells). (**C**) Quantification of Oil Red O staining of cultured human cells isolated from healthy kidney tissue and clear-cell RCC tissue treated with siRNA against HIF-1α or VLDL-R (*n* = 10, *p≤0.001 vs. control siRNA normal cells, †*p*≤0.05 vs. control siRNA clear-cell RCC cells). (**D**) Quantification of fluorescently internalized DiI-labeled lipoproteins in cultured human cells isolated from healthy kidney tissue and clear-cell RCC tissue treated with siRNA against HIF-1α or VLDL-R (*n* = 5, *p≤0.05 vs. control siRNA normal cells, †*p*≤0.05 vs. control siRNA clear-cell RCC cells). Data are shown as mean ± SEM.

We also investigated the involvement of HIF-1α in the upregulation of VLDL-R expression in clear-cell RCC. As expected, the clear-cell RCC cells showed increased HIF-1α protein levels (Figure S1A) and HIF-1α activity as shown by increased mRNA expression of known HIF-1α-responsive genes (Figure S1B–D). Partial knockdown of HIF-1α by siRNA (Figure S1) significantly reduced the expression of both VLDL-R mRNA and protein (Figure 2A and B). Furthermore, knockdown of either HIF-1α or VLDL-R by siRNA significantly reduced the increased lipid accumulation observed in clear-cell RCC cells (Figure 2C). These data suggest that HIF-1α mediates increased VLDL-R overexpression in clear-cell RCC cells, which promotes increased lipid accumulation.

In addition, we assessed whether the expression of other lipid receptors and/or mediators of lipid metabolism was affected by VLDL-R siRNA knockdown or modified in clear-cell RCC cells compared with normal kidney cells. We found that knockdown of the VLDL-R did not influence the expression of the lipoprotein receptors macrophage scavenger receptor 1 (MRS1) and scavenger receptor B1 (SCARB1), the lipid efflux receptors ATP-binding cassette subfamily A member 1 (ABCA1) and ATP-binding cassette subfamily G member 1 (ABCG1), the fatty acid transport protein CD36 or lipoprotein lipase (LPL) (Figure S2A–G), ruling out off-target effects and compensatory regulations. In addition, VLDL-R siRNA knockdown did not influence the activity of ACAT in clear-cell RCC cells (Figure S2H). As has been previously shown, LDL-R expression was significantly lower in clear-cell RCC (Figure S2C), and SCARB1 expression was increased in clear-cell RCC cells compared with normal kidney cells (Figure S2B) [11].

To investigate if VLDL-R overexpression changed the phenotype of primary cells from normal kidney tissue, we transfected these control cells with a vector encoding VLDL-R. Although we observed an increase in VLDL-R expression (1.39±0.13-fold; *n* = 4; *p*<0.05 vs control vector) and a similar increase in lipid accumulation (2.2±0.2-fold; *n* = 4; *p*<0.05 vs control vector), these increases were much smaller than those observed in clear-cell RCC cells (Figure 1C, D). Transfection with a VLDL-R vector did not affect mRNA expression of HIF-1α-responsive genes (data not shown), but this is not surprising because we propose that the constitutive activation of HIF-1α in clear-cell RCC cells promotes VLDL-R expression and not the other way round.

To investigate if the lipid accumulation in clear-cell RCC cells is dependent on increased lipid uptake mediated by the VLDL-R, we incubated the isolated primary kidney cells with fluorescently labeled lipoprotein particles. We showed that the uptake of fluorescently labeled lipoproteins was significantly higher in clear-cell RCC cells compared with control cells (Figure 2D). Partial knockdown of either HIF-1α or VLDL-R using siRNA significantly reduced the uptake of lipoproteins in primary clear-cell RCC cells but not in primary control cells (Figure 2D). These data support the concept that the pathological increase of HIF-1α in clear-cell RCC cells upregulates VLDL-R, which in turn mediates the increased uptake and accumulation of lipids.

Although the levels of cholesteryl esters were 230-fold higher in the clear-cell RCC biopsies compared with normal kidney tissue, the triacylglycerol content was only increased 8-fold (Table 1). Lipoproteins that bind to the VLDL-R contain mainly triglycerides, but they also contain cholesterol and cholesteryl esters [13], [14], [15]. It is likely that clear-cell RCC cells utilize the triglycerides for energy consumption [6], but accumulate cholesteryl esters due to increased ACAT activity [8]. Thus, our results indicate that the characteristic lipid accumulation in clear-cell RCC cells is, at least partly, dependent on an increase in the uptake of plasma lipoproteins mediated by HIF-1α-induced overexpression of VLDL-R. These results explain the morphological characteristic of clear-cell RCC. The VLDL-R could potentially be used as a biomarker of clear-cell RCC and it plausible that novel therapeutics can be developed that deliver drugs specifically to clear-cell RCC via uptake through the VLDL-R.

## Methods

### Kidney biopsies and preparation of primary kidney cells

Tissue from clear-cell RCC was obtained from nephrectomies from six patients for biopsy experiments and from ten further patients for isolation of primary cells. Non-cancerous cortical tissue farthest from the tumor in the same patients was used as control. Areas for collection were chosen by an experienced pathologist. Biopsies were frozen in liquid nitrogen until analysis. Cells were isolated as described previously [16] and plated in DMEM supplemented with 10% FCS, 1% PEST and 1% L-glutamine.

The experimental protocol was approved by the Lund Regional Ethics Committee and performed according to the Declaration of Helsinki. All patients gave written informed consent.

*Lipid accumulation.* Tissue sections and cells were stained with Oil Red O as described previously [12]. Briefly, frozen kidney biopsies were cryosectioned in 4 µm slices, transferred to polysine glass slides, and stained with Oil Red O. The slides were mounted with mowiol, photographed with a Zeiss Axioplan microscope, and the area of Oil Red O staining per tissue area was determined as described.

### Lipid analysis

ipidomics analysis was performed on biopsies from six patients. Tissue (50–100 mg) was homogenized using a combination of Precellys 24 homogenizer (Bertin Technologies, Montigny-le-Bretonneux, France) and Mixer Mill equipment (Retsch, Haan, Germany). Lipids were extracted according to Folch et al [17]. The lipids were quantified using a combination of straight-phase HPLC with evaporative light scattering detection [18] and mass spectrometry as described previously [19], [20], [21]. See Methods S1 for further details.

### Immunohistochemistry

Sections of formalin-fixed and paraffin-embedded (FFPE) human renal tissue, thickness 4 µm, were deparaffinized using xylene followed by graded ethanol for rehydration. Boiling in 10 mM citrate buffer at pH 6 was performed as antigen retrieval. Sections were incubated with VLDL-R antibodies (R&D Systems), which were detected using the EnVision system and DAKO Techmate 500, according to the manufacturer's instructions (DAKO, Glostrup, Denmark). Chromogens used were diaminobenzidine (black/brown) and hematoxylin as counterstaining.

### Analysis of mRNA and protein expression

Total RNA was isolated from cells using the RNeasy Mini Kit (Qiagen) and cRNA was synthesized using the cDNA archive kit (Applied Biosciences). Results were analyzed with Taqman and normalized against *18S* mRNA. Immunoblots were performed as described previously [12].

### Lipoprotein uptake

Primary kidney cells were incubated with lipoproteins fluorescently labeled with 1,1′-dioctadecyl-1-3,3,3′,3′-tetramethylindocarbocyanine perchlorate (DiI) (10 µg/ml) for 3 h as described previously _ENREF_13 [12]. Micrographs were captured by confocal microscopy and lipoprotein uptake was determined by measuring the intracellular fluorescent area of each cell.

*Transfection.* Primary human kidney cells were transfected with siRNAs (Applied Biosciences) or a vector expressing VLDL-R using Lipofectamine 2000 (Invitrogen) 48 h before analysis.

### ACAT activity

ACAT1 activity was measured as described previously [22].

## Supporting Information

Figure S1 **HIF-1α expression and activity are increased in clear-cell RCC.** (A) Quantification of immunoblot against HIF-1α with β-actin as loading control from cultured human cells isolated from healthy kidney tissue and clear-cell RCC tissue treated with siRNA against HIF-1α or VLDL-R (*n* = 10, *p≤0.05 vs. control siRNA normal cells, †*p*≤0.05 vs. control siRNA clear-cell RCC cells). (B, C, D) Quantification of mRNA expression of HIF-1α-responsive genes [(B) vascular endothelial growth factor α (VEGFα), (C) glyceraldehyde 3-phosphate dehydrogenase (GAPDH) and (D) erythropoeitin (EPO)] normalized to 18S mRNA from cultured human cells isolated from healthy kidney tissue and clear-cell RCC tissue transfected with HIF-1α or control siRNA (*n* = 10, *p≤0.05 vs. control siRNA normal cells, †*p*≤0.05 vs. control siRNA clear-cell RCC cells).
(TIF)

Figure S2 **VLDL-R knockdown does not affect the expression of other lipid receptors or mediators of lipid metabolism in clear-cell RCC cells.** (A–G) Quantification of mRNA expression of (A, B) scavenger receptors MSR1 and SCARB1, (C) LDL-R, (D, E) lipid efflux receptors ABCA1 and ABCG1, (F) fatty acid transport protein CD36 and (G) LPL normalized to 18S mRNA from cultured human cells isolated from healthy kidney tissue and clear-cell RCC tissue transfected with VLDL-R or control siRNA (*n* = 10, *p≤0.05 vs. control siRNA normal cells). (H) ACAT activity in clear-cell RCC cells transfected with control or VLDL-R siRNA (*n* = 4).
(TIF)

Table S1 **Cholesteryl ester species in human tissue sections from normal kidney tissue and clear-cell RCC tissue (CCRCC).**
(DOC)

Methods S1 Supplemental Methods.
(PDF)
