# Supplementary figures and images for: Increased Expression of the Very Low-Density Lipoprotein Receptor Mediates Lipid Accumulation in Clear-Cell Renal Cell Carcinoma
Source: PLoS One. 2012 Nov 19;7(11):e48694. doi: 10.1371/journal.pone.0048694 (PMC3501495; doi:10.1371/journal.pone.0048694)

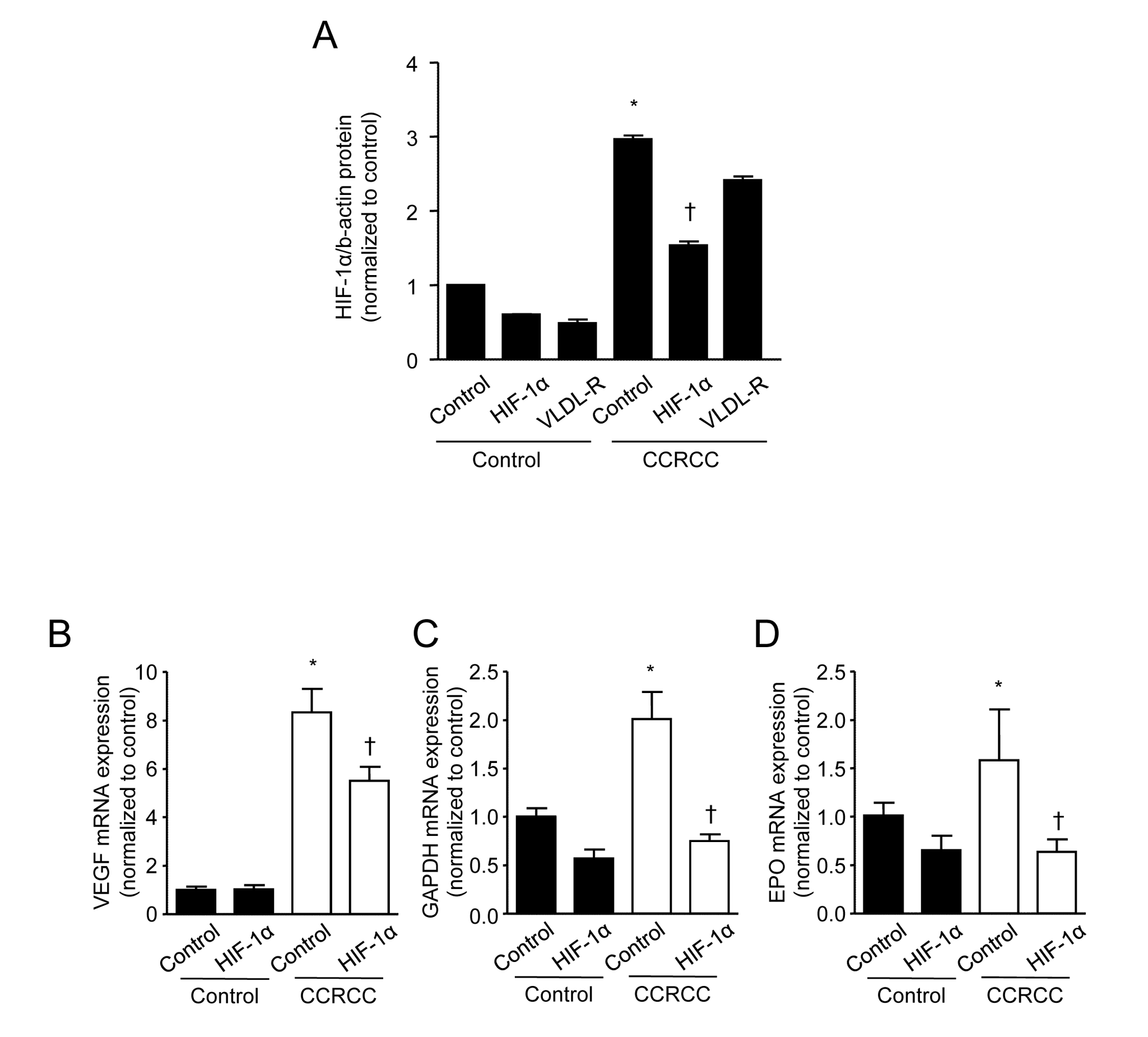

Supplement: Figure S1 — HIF-1α expression and activity are increased in clear-cell RCC. (A) Quantification of immunoblot against HIF-1α with β-actin as loading control from cultured human cells isolated from healthy kidney tissue and clear-cell RCC tissue treated with siRNA against HIF-1α or VLDL-R (n = 10, *p≤0.05 vs. control siRNA normal cells, †p≤0.05 vs. control siRNA clear-cell RCC cells). (B, C, D) Quantification of mRNA expression of HIF-1α-responsive genes [(B) vascular endothelial growth factor α (VEGFα), (C) glyceraldehyde 3-phosphate dehydrogenase (GAPDH) and (D) erythropoeitin (EPO)] normalized to 18S mRNA from cultured human cells isolated from healthy kidney tissue and clear-cell RCC tissue transfected with HIF-1α or control siRNA (n = 10, *p≤0.05 vs. control siRNA normal cells, †p≤0.05 vs. control siRNA clear-cell RCC cells). (TIF) [file pone.0048694.s001.tif]

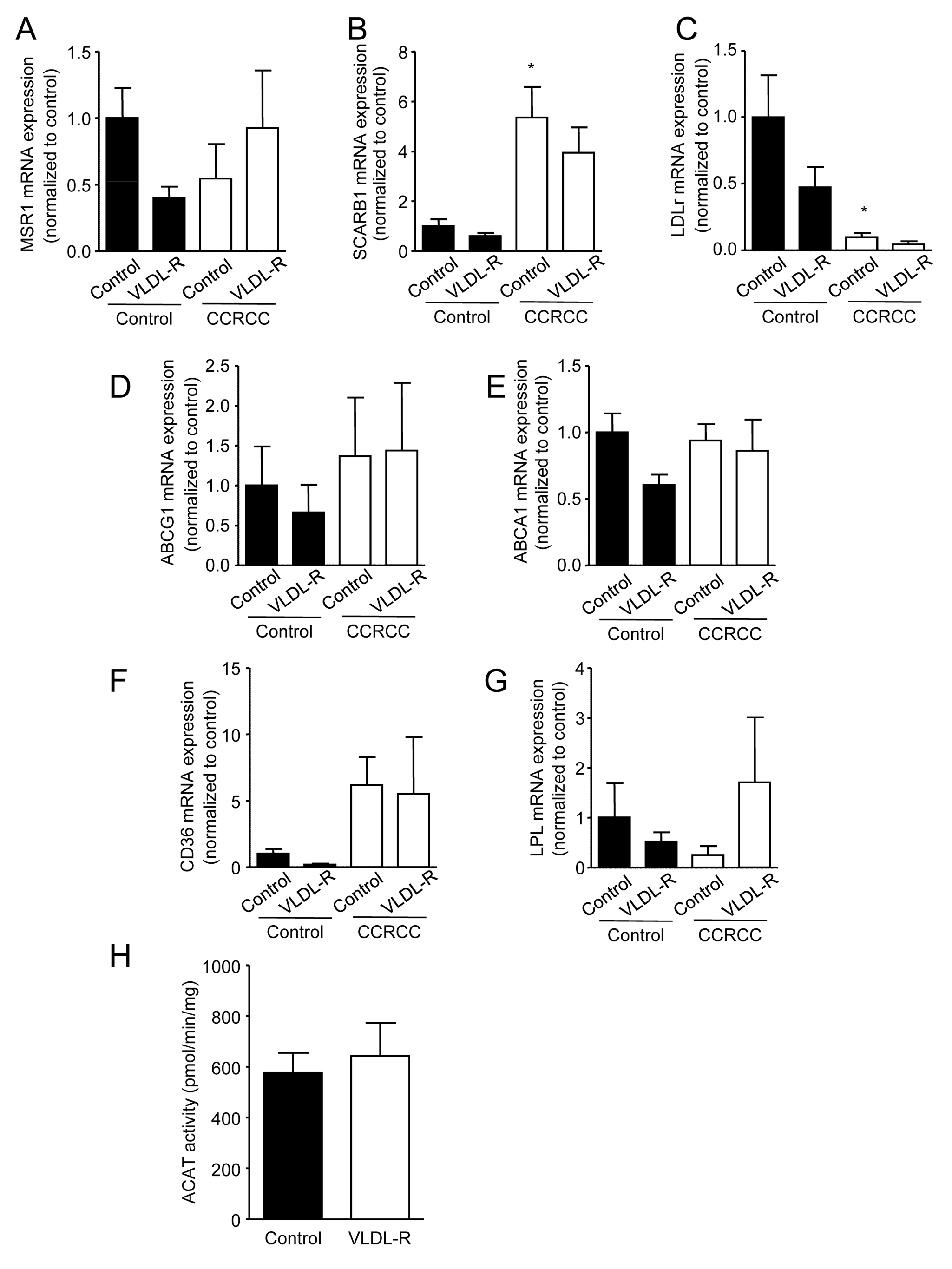

Supplement: Figure S2 — VLDL-R knockdown does not affect the expression of other lipid receptors or mediators of lipid metabolism in clear-cell RCC cells. (A–G) Quantification of mRNA expression of (A, B) scavenger receptors MSR1 and SCARB1, (C) LDL-R, (D, E) lipid efflux receptors ABCA1 and ABCG1, (F) fatty acid transport protein CD36 and (G) LPL normalized to 18S mRNA from cultured human cells isolated from healthy kidney tissue and clear-cell RCC tissue transfected with VLDL-R or control siRNA (n = 10, *p≤0.05 vs. control siRNA normal cells). (H) ACAT activity in clear-cell RCC cells transfected with control or VLDL-R siRNA (n = 4). (TIF) [file pone.0048694.s002.tif]
