# Supplementary material for: Increased Expression of the Very Low-Density Lipoprotein Receptor Mediates Lipid Accumulation in Clear-Cell Renal Cell Carcinoma
Source: PLoS One. 2012 Nov 19;7(11):e48694. doi: 10.1371/journal.pone.0048694 (PMC3501495; doi:10.1371/journal.pone.0048694)
Supplement: Table S1 — Cholesteryl ester species in human tissue sections from normal kidney tissue and clear-cell RCC tissue (CCRCC). (DOC) [file pone.0048694.s003.doc]

**Table S1**. Cholesteryl ester species in human tissue sections from normal kidney tissue and clear-cell RCC tissue (CCRCC).

| **Composition** | **Control**  **(n = 6)** | **CCRCC**  **(n = 6)** | ***p* value** |
| --- | --- | --- | --- |
| C 14:0 | 0.8 ± 0.1 | 2.0 ± 1.8 | ns |
| C 16:1 | 4.0 ± 1.0 | 6.7 ± 5.4 | ns |
| C 16:0 | 10.4 ± 0.4 | 9.2 ± 1.7 | ns |
| C 18:3 | 1.9 ± 0.4 | 1.3 ± 0.4 | 0.0188 |
| C 18:2 | 39.4 ± 7.0 | 13.3 ± 5.4 | < 0.0001 |
| C 18:1 | 28.8 ± 5.7 | 49.3 ± 12.4 | 0.0035 |
| C 18:0 | 1.3 ± 0.3 | 4.6 ± 0.4 | < 0.0001 |
| C 20:5 | 2.6 ± 1.1 | 1.3 ± 0.6 | 0.0273 |
| C 20:4 | 8.4 ± 1.9 | 3.4 ± 2.0 | 0.0008 |
| C 20:3 | 1.3 ± 0.5 | 3.0 ± 2.0 | ns |
| C 20:2 | 0.4 ± 0.3 | 2.3 ± 1.2 | 0.0031 |
| C 20:1 | 0.6 ± 0.5 | 3.8 ± 1.1 | < 0.0001 |

All values are mol%. Data are mean ± SEM.
