# Supplementary material for: Increased Expression of the Very Low-Density Lipoprotein Receptor Mediates Lipid Accumulation in Clear-Cell Renal Cell Carcinoma
Source: PLoS One. 2012 Nov 19;7(11):e48694. doi: 10.1371/journal.pone.0048694 (PMC3501495; doi:10.1371/journal.pone.0048694)
Supplement: Methods S1 — Supplemental Methods. (PDF) [file pone.0048694.s004.pdf]

SUPPLEMENTAL METHODS

Lipid analysis. Lipidomics analysis was performed on biopsies from six patients. Tissue (50-100 mg) was homogenized with ceramic beads in 500 μl methanol using the Precellys 24 homogenizer (Bertin Technologies, Montigny-le-Bretonneux, France) followed by mixing at 25 Hz for 10 min using Mixer Mill equipment (Retsch, Haan, Germany). The tissue homogenate was transferred to glass tubes and the homogenization tubes were washed with 500 μl methanol. Internal standards (PC 17:0/17:0, PE 17:0/17:0, DAG 17:0/17:0, LPC 17:0 CER 17:0 and SM 17:0) were added dissolved in 2000 μl chloroform and the lipids were extracted according to Folch et al.[1] The lipid extract was evaporated under a stream of nitrogen and reconstituted in chloroform:methanol [2:1 v:v] and stored at -20 C until analysis.

Cholesteryl esters, triacylglycerols and free cholesterol were quantified using straight-phase HPLC with evaporative light scattering detection according to previous work.[2] Phospholipids and sphingomyelin were analyzed as described[3, 4] using direct infusion on a QTRAP 5500® mass spectrometer (AB Sciex, Concord, Canada) equipped with a robotic nanoflow ion source NanoMate Triversa (Advion Biosciences, Ithaca, NY, USA). Cholesteryl ester profiles were attained in precursor ion scanning mode using the same equipment.[5] Mass spectrometry data files were processed using Lipid Profiler™.[3] Diacylglycerols were purified using straight-phase HPLC prior to analysis using precursor ion scanning as described previously.[6] Identified lipids were quantified by normalizing against their respective internal standard and protein content. The ether lipids of phosphatidylcholine and phosphatidylethanolamine were quantified using their acyl analogs.

After purification using straight-phase HPLC, the ceramides were analyzed using reversed phase HPLC coupled to a triple quadrupole Quattro Premiere mass spectrometer (Waters, Milford, USA). The HPLC system consisted of two solvents: solvent A was water:acetoniltril (63:37; v/v) with 0.1% formic acid and solvent B was acetonitrile:isopropanol (1:1; v/v). The ceramides were separated on an XBridge C-18 2.5μ column (2.1 x 50 mm) (Waters, Milford, USA) with a linear gradient from 80%B-100%B for 7 min. The gradient was then returned to initial conditions and the column was equilibrated for 7 min to give a total runtime of 14 min. The flow rate was set to 0.3 ml/min. Ceramide species were quantified using external standards.

Supplemental Figure 1. HIF-1α expression is increased in clear-cell RCC. Quantification of immunoblot against HIF-1α with β-actin as loading control from cultured human cells isolated from healthy kidney tissue and clear-cell RCC tissue treated with siRNA against HIF-1α or VLDL-R (*n* = 10, *$p \leq 0.05$ vs. control siRNA normal cells, †$p \leq 0.05$ vs. control siRNA clear-cell RCC cells).

Supplemental Table 1. Cholesteryl ester species in human tissue sections from normal kidney tissue and clear-cell RCC tissue (CCRCC).

Composition	Control (n = 6)	CCRCC (n = 6)	*p* value
C 14:0	0.8 ± 0.1	2.0 ± 1.8	ns
C 16:1	4.0 ± 1.0	6.7 ± 5.4	ns
C 16:0	10.4 ± 0.4	9.2 ± 1.7	ns
C 18:3	1.9 ± 0.4	1.3 ± 0.4	0.0188
C 18:2	39.4 ± 7.0	13.3 ± 5.4	< 0.0001
C 18:1	28.8 ± 5.7	49.3 ± 12.4	0.0035
C 18:0	1.3 ± 0.3	4.6 ± 0.4	< 0.0001
C 20:5	2.6 ± 1.1	1.3 ± 0.6	0.0273
C 20:4	8.4 ± 1.9	3.4 ± 2.0	0.0008
C 20:3	1.3 ± 0.5	3.0 ± 2.0	ns
C 20:2	0.4 ± 0.3	2.3 ± 1.2	0.0031
C 20:1	0.6 ± 0.5	3.8 ± 1.1	< 0.0001

All values are mol%. Data are mean ± SEM.

REFERENCES

1. Folch, J, Lees, M, Stanley, GHS: A simple method for the isolation and purification of total lipides from animal tissues. *J Biol Chem,* 226**:** 497-509, 1957.
2. Homan, R, Anderson, MK: Rapid separation and quantitation of combined neutral and polar lipid classes by high-performance liquid chromatography and evaporative light-scattering mass detection. *J Chromatogr B Biomed Sci Appl,* 708**:** 21-26, 1998.
3. Ejsing, CS, Duchoslav, E, Sampaio, J, Simons, K, Bonner, R, Thiele, C, Ekroos, K, Shevchenko, A: Automated identification and quantification of glycerophospholipid molecular species by multiple precursor ion scanning. *Anal Chem,* 78**:** 6202-6214, 2006.
4. Ekroos, K, Chernushevich, IV, Simons, K, Shevchenko, A: Quantitative profiling of phospholipids by multiple precursor ion scanning on a hybrid quadrupole time-of-flight mass spectrometer. *Anal Chem,* 74**:** 941-949, 2002.
5. Liebisch, G, Binder, M, Schifferer, R, Langmann, T, Schulz, B, Schmitz, G: High throughput quantification of cholesterol and cholesteryl ester by electrospray ionization tandem mass spectrometry (ESI-MS/MS). *Biochim Biophys Acta,* 1761**:** 121-128, 2006.
6. Ejsing, CS, Sampaio, JL, Surendranath, V, Duchoslav, E, Ekroos, K, Klemm, RW, Simons, K, Shevchenko, A: Global analysis of the yeast lipidome by quantitative shotgun mass spectrometry. *Proc Natl Acad Sci U S A,* 106**:** 2136-2141, 2009.

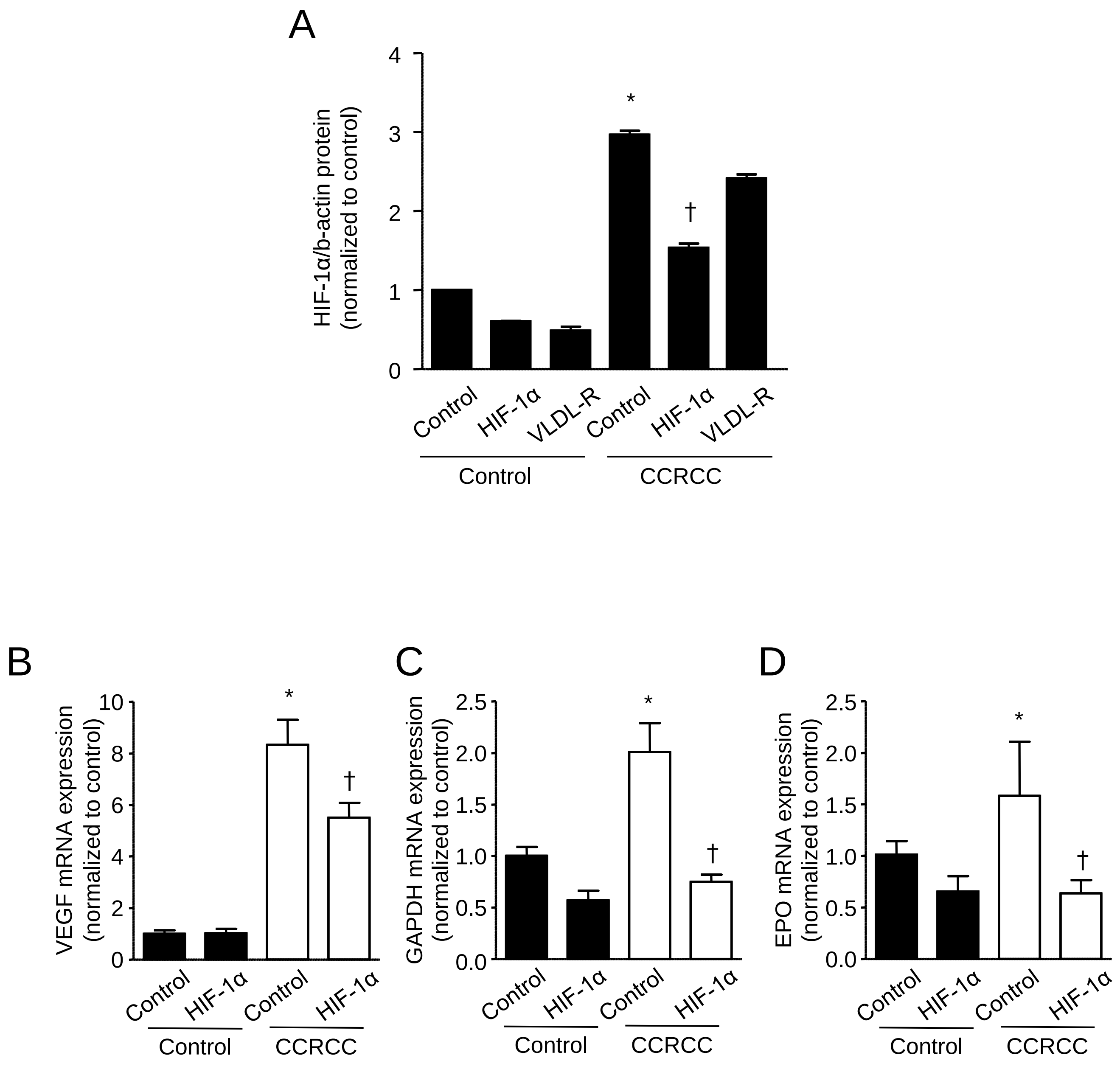

Supplemental Figure 1

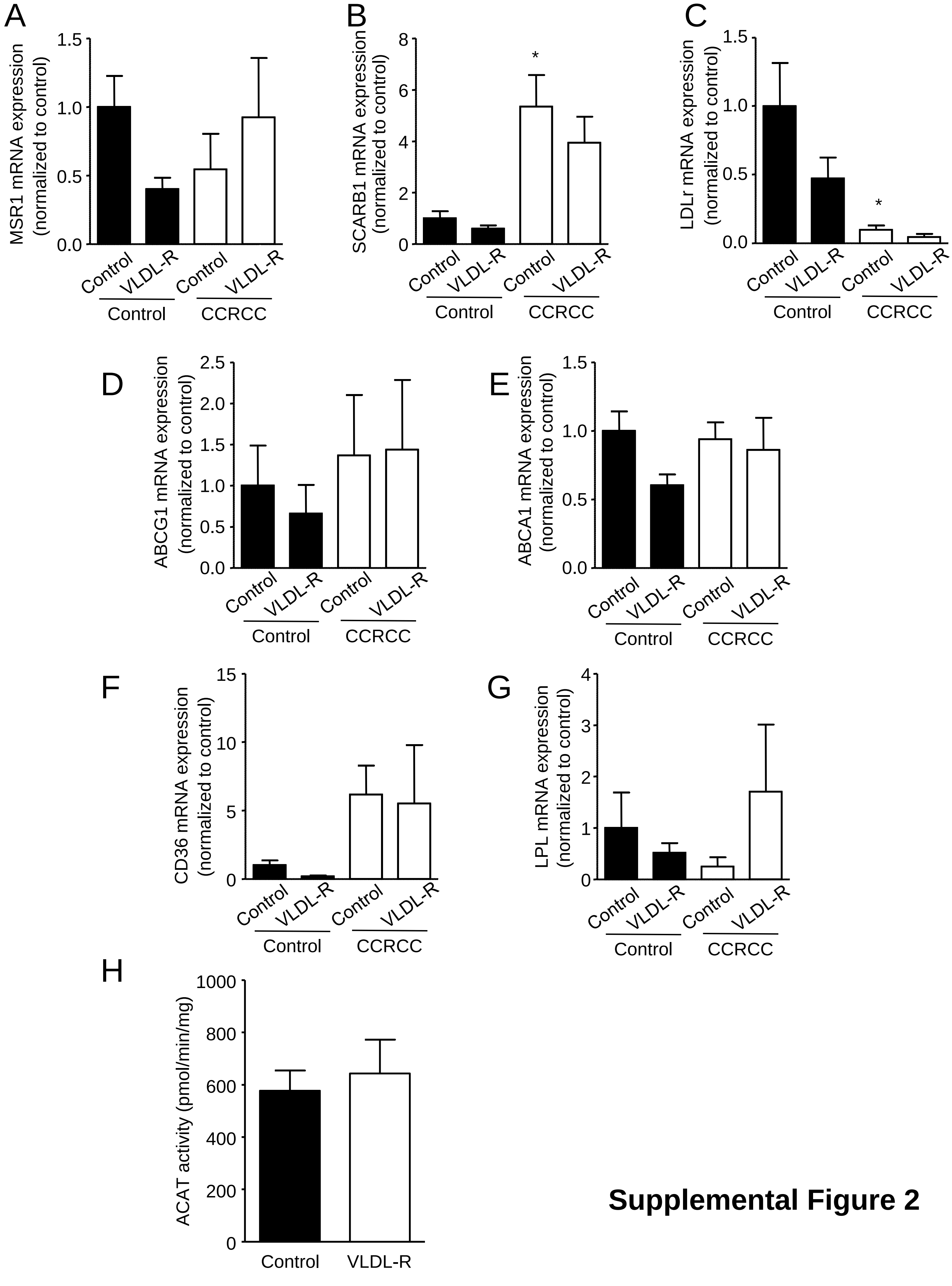

Supplemental Figure 2
